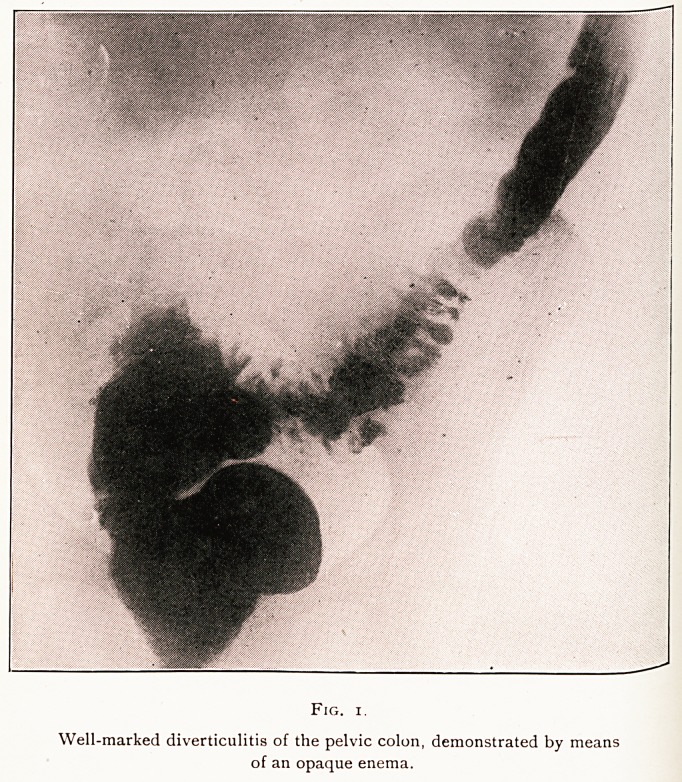# Diverticulitis of the Pelvic Colon
1A Paper read at a Meeting of the Bath and Bristol Branch of the British Medical Association on Wednesday, February 27th, 1924.


**Published:** 1924-07

**Authors:** C. Gordon-Watson

**Affiliations:** Surgeon to St. Bartholomew's and St. Mark's Hospitals


### DIVERTICULITIS OF THE PELVIC COLON.[1]

BY

SIR C. GORDON-WATSON, K.B.E., C.M.G., F.R.C.S.,
*Surgeon to St. Bartholomew's and St. Mark's Hospitals.*

---

In recent years our attention has been focussed on stagnation in the colon and the evil results that ensue. In this direction Sir Arbuthnot Lane has done much to stimulate interest, to excite admiration, and indeed to inspire awe into the hearts of not a few of us. The late Father Bernard Vaughan was not more scathing on "The Sins of Society" than Lane has been on "The Sins of the Colon." And who shall say that these strictures are not without good cause? In recent years the punishment for a colon steeped in iniquity has been total extirpation. There is, at the moment, a movement afoot to abolish capital punishment for the worst sins of society; at the same time we learn that Sir Arbuthnot is in favour of abolishing the extreme penalty for the errant colon. He now believes that surgical relief of stasis can be secured by "careful freeing of acquired adhesions of the pelvic colon to the iliac fossa." Indeed, he states in an address recently published in the *British Medical Journal* that he has "on many occasions derived the same advantage by this simple operation that he had

[1] A Paper read at a Meeting of the Bath and Bristol Branch of the British Medical Association on Wednesday, February 27th, 1924.

previously had from colectomy at a considerably greater risk."

Lane has drawn a remarkable clinical picture of the mechanical and toxic sequelæ, which in his opinion follow the development of newly-formed peritoneal bands that anchor the overloaded pelvic colon to the iliac fossa, and by contraction cause what he describes as "the first and last kink."

The determining factors in the formation of diverticula of the colon are not yet satisfactorily established. It is probable that increased pressure within the colon due to constipation and flatulent distension is the most important factor, and it is more than probable that the inflammatory conditions which often result from fæcal retention in these pouches must be laid at the door of intestinal stasis.

Constipation and flatulence are not the whole story; if they were we should expect to meet with these pressure pouches far more often. Diverticulitis is rarely met with under forty and far more often over fifty, and in the majority of cases extraperitoneal fat is present in excess. It may be said with much truth that the victims of diverticulitis are in most instances past the meridian of life, of sedentary habits, fat, flabby and flatulent.

Willey of Sheffield believes that there is a close connection between the emotions and pathological conditions of the colon, and regards a sensitive and impassioned temperament as a powerful predisposing factor in diverticulitis.

With the gradual increase of fat around the bowel there is progressive atrophy of the unstriated muscle.

Normally, as we know from radiograms, the colon during contraction presents alternate rings of contraction and sacculation. When subject to long-continued back pressure an overloaded and flatulent colon with fat-laden and atrophied muscle must, during straining at stool, be prone to yield in the sacculated portions at the weakest points. We know that diverticula once formed sometimes perforate during the act of straining at stool or from some sudden exertion.

As Hamilton Drummond has shown, these weak spots occur where the circular vessels perforate the muscular coat to reach the mucosa.

These weak spots occur on either side between the lateral tænia and the mesenteric border, and it is along these lines that diverticula most commonly occur.

The diverticula are small herniæ of the mucosa through the muscular coats, which gradually enlarge, and as they increase in size tend to become flask-shaped and bottle-necked and to act as traps for fæcal matter.

Though the exact pathology of the origin of diverticula in the colon is uncertain, the pathology of diverticulitis, *i.e.* of the inflammatory phenomena which result from the retention of fæcal matter in these hernial pouches, is thoroughly straightforward, and bears so close an analogy to the many-sided phenomena of appendix inflammation as to justify the description of "left-sided appendicitis."

Fæcal stagnation may be followed by the formation of concretions, concretions by ulceration, ulceration by perforation, perforation by either general peritonitis or more often by a localised abscess and perhaps a fæcal fistula. In other instances inflammation inside a diverticulum results not in perforation but in inflammation outside which is chronic from the first, and gives rise to peridiverticular fibrosis, fibrosis to tumour formation and stenosis, stenosis to chronic obstruction and a mimicry of carcinoma.

As Maxwell Telling so aptly says, "Given the formation of multiple herniæ of the mucosa, every secondary process that occurs may be logically deduced *a priori* by general pathological comparison."

Inflammation in and around these acquired diverticula thus produces three main clinical conditions: (1) acute manifestations associated with perforation which cannot be distinguished from the acute manifestations of appendicitis except as regards the left-sided symptoms; (2) a subacute suppurative condition analogous to the appendix abscess and distinguished by a tendency to fæcal fistula or vesico-colic fistula; (3) the hyperplastic type which so closely simulates carcinoma in that a tumour forms which frequently causes obstruction.

While recognising the many phases of diverticular inflammation it is important to remember that diverticula frequently exist and give rise to no symptom at all, and this is more likely to be the case when the pouches are tubular and wide at the neck. In recent years, since I have been on the look out for them, I have met with innocent diverticula on a few occasions during an abdominal operation. Indeed, they are easily missed when searched for at autopsies, both from without and within, though when they contain solid fæcal matter hard lumps may often be felt in the appendices epiploicæ. These small pouches may readily escape detection, because they occur in fat-laden bowel and usually project into the fatty appendices epiploicæ. This condition has been termed diverticulosis as opposed to diverticulitis.

When there is a considerable inflammation round the bowel the diverticula may only be recognised after careful dissection.

The openings within the bowel lie between the rugæ, and are often so small as not to be recognisable until the mucosa is put on the stretch.

Drummond examined the colon in 500 post-mortems and found diverticula in 22 cases, and it is interesting to note that in four of them there were also diverticula in the small intestine and in one case a diverticulum of the bladder, facts which are suggestive of some congenital defect in the unstriated muscle.

In 1,000 radiograms of the intestine Spriggs only recognised diverticula in six, but there was no special investigation for diverticula. The diagnosis of diverticulitis usually depends on radiographic evidence.

As in other conditions, with perfect X-rays after an opaque meal we may get positive evidence which makes the diagnosis a certainty, but the reverse by no means holds good. The appearance of clear-cut diverticula, when seen in a skiagram, is unmistakable, and the diagnosis is readily confirmed when the opaque pockets remain visible after the remainder of the bowel has been emptied.

Evidence of diverticula in the pelvic colon may be missed unless the patient is examined when the bulk of the meal is in this region, and again examined when the pelvic colon has been emptied. If the diverticula are numerous positive evidence may be expected; if only a few are present, and these are filled with fæcal material, or are perhaps so situated as to be obscured by the main outline of the bowel (*i.e.* not shown in profile), they may escape observation.

Positive evidence in doubtful cases is more likely to be obtained by an enema, which may distend the pockets and also show up any filling defect in the main lumen.

Belladonna given prior to an enema may by relaxing the muscular coats increase the patency of the pockets.

Irregularities in the function of the colon present great difficulty as regards diagnosis. When a middle-aged patient presents himself with the story that he is much worried with flatulent distension and irregularity in the action of the bowels the practitioner may be faced with a troublesome problem. In the absence of a palpable growth in the rectum he does not know whether to assume that the disturbance of function is due to the "first and last kink," *i.e.* stasis, or is an inflammatory condition, *i.e.* a colitis resulting from errors of diet and unhealthy habits, or whether he is up against diverticulitis or an early carcinoma of the colon.

It is reasonable to hope that diagnosis may be simplified by advances in radiography and chemical pathology.

Fortunately, diverticulitis is one of the rarities of practice. The acute manifestations, if they seldom receive, seldom demand exactitude in diagnosis; they take their humble place among the acute abdomens. For better or for worse, they run their luck.

More interest, I think, attaches to the subacute and chronic cases which simulate malignant stricture, and I propose to give you my personal experiences with this type of case. An exact diagnosis may sometimes be made with the sigmoidoscope, though more often with opaque radiography; a probable diagnosis may be possible from the clinical history.

In most instances the area involved is beyond the reach of the sigmoidoscope, frequently because owing to adhesions the sigmoidoscope cannot be passed to the full extent.

Exceptionally the mouths of diverticula may be seen with the sigmoidoscope, and an absolute diagnosis established.

In June of last year a stout lady, aged 64, was brought to me with a history that for some months she had been greatly troubled with flatulent distension, and with small, frequent teasing motions usually loose but often containing small "rabbit-like" hard débris. Occasionally she had passed blood-stained mucus. There had been no loss of weight, and her appearance did not suggest malignant disease. On examination with the sigmoidoscope I observed at 20 cm. the mouths of two diverticula. They were visible only on inflation, *i.e.* they could be seen to open up with each puff of the inflator. The patient, unfortunately, took fright either at the sigmoidoscope or at the diagnosis, and I heard no more of her.

It should be noticed that the passage of blood and mucus so diagnostic of new growth occurred in this case. This is exceptional, and is usually an important point in differential diagnosis. It may be that in this case the two conditions were combined, as is sometimes the case. Carcinoma may arise, or, as it were, settle down in the midst of a diverticulitis. In other instances diverticula form above a carcinomatous stricture. Although it has been my lot to deal with a large number of colon carcinomata, I have not yet met with diverticula in this association. There are, however, several recorded cases of both these associated conditions, and I have seen specimens illustrating them. Grey Turner operated on a case in which a perforation of a diverticulum occurred above a carcinoma.

One would expect the formation of diverticula to be not uncommon behind a stricture of the colon if increased colonic tension is the main factor in their production.

The fact that the opposite is the case makes me think that we are still lacking in full information as to their origin.

The first case of diverticulitis that I operated on was in 1911 at St. Bartholomew's, and this was a case which was almost certainly secondary to stricture of the rectum. The patient was a young woman of 26 when I operated on her, one of the youngest cases on record. I described the case at the time as "Pericolitis Sinistra with Sacculations." I was then ignorant of the term diverticulitis. At the age of 18 she was operated on (posterior proctotomy) for stricture of the rectum by Mr. Harrison Cripps. Five years later she was treated by bougies for recurrent stricture. Three years after this I found a tight, fibrous stricture of the lower part of the rectum and opened the abdomen to perform colostomy. I found the pelvic colon adherent to the uterus greatly inflamed with numerous appendices epiploicæ, many of which contained saccules of mucous membrane. Owing to the contracted mesocolon and a very friable intestine I was unable to perform colostomy until I had divided the bowel and closed the lower end.

Fig. 1.

Well-marked diverticulitis of the pelvic colon, demonstrated by means of an opaque enema.

Radiographic evidence may be quite conclusive. The following case is an example :—

A woman, aged 56 (first seen January 1st, 1923), robust looking and well covered, complained of frequent offensive stools with troublesome colicky pains and the passage of a good deal of mucus in the stools. For the past few years she has suffered in a minor degree from these attacks, which have been followed by quiescent periods. Recently there has been more difficulty in getting the bowel empty in spite of increasing doses of aperients.

A barium enema showed " well-marked filling defect in the pelvic colon," not the filling defect suggestive of an annular stricture due to carcinoma, but a more diffuse stenosis involving several inches of the bowel. (Fig. 1.)

Radiographs taken after evacuation of the contents of the bowel showed the classical multiple opaque blobs so characteristic of diverticulitis.

Operation revealed not only multiple diverticula but extensive peridiverticular inflammation. There were many adhesions between the pelvic colon and the iliac fossa. The inflamed area was so extensive that a short circuit between transverse and pelvic colon was not feasible. The patient was opposed to colostomy unless considered absolutely essential. There seemed to be some risk that acute symptoms might intervene from perforation of a diverticulum.

Invagination of diverticula has been practised when perforation has threatened, but is only practicable when the diverticula are few and blessed with wide necks.

I eventually separated the pelvic colon from its parietal adhesions, and then wrapped a superabundant great omentum round the involved area, stitching the free edge of the omentum to the outer layer of the mesentery.

In this way I hoped to avoid any general peritoneal infection should a perforation occur, and at the same time by relief of adhesions to increase the mobility and to improve the functional ability of the pelvic colon. The after treatment in this case has consisted in the regular use of paraffin internally and daily enemata to prevent stagnation in the colon. Radiograms taken a year after operation show that the stenosis remains. The patient is involved in a daily toilet which takes considerable time but secures comfort for the remainder of the day. I think the toilet would be less strenuous if she had a colostomy opening above the involved area.

In many instances a sausage-shaped tumour in the left iliac fossa may be felt per abdomen, and a diagnosis assumed when the clinical picture is strongly against malignant disease.

The following case is an example of this, and presents many features of interest :—

A clergyman, aged 46, was seen by me in 1914 with the following history. Twenty years ago he had an attack of jaundice. For the last fifteen years he had had dyspepsia, taking the form of acid eructations and pyrosis, in the strict sense of the word, after food. About the same time he began to have difficulty with the bowels ; after an action he would still feel that there was something there, and there might be as many as four or five small actions before he was comfortable. Gradually the bowels became so capricious in their action that he used to take some astringent when he had to perform some public duty. About two to three years ago he began to pass blood and mucus with the stools. A few months ago an X-ray examination with bismuth was made, but revealed no structural change in stomach or colon ; the marked distension of the stomach on giving sodium bicarbonate confirmed the diagnosis of hyperchlorhydria, however. He was stout and not anæmic. Physical examination revealed nothing till the left iliac fossa was reached. Here, even after the bowel had been cleared out by enemata, a sausage-shaped, definitely tender swelling was felt. The abdomen was opened, and the diseased bowel, about six inches in length, was brought to the surface and fixed. Eight days later (June, 1914) I excised the affected area and established a temporary colostomy. A year afterwards, in 1915, during my absence at the war, the colostomy was closed by Mr. Lockhart Mummery. Two years afterwards (1917) there were recurrent symptoms of partial obstruction. The transverse was now anastomosed to the pelvic colon at the junction with the rectum, and a radiograph taken a year later, in 1918, shows the anastomosis in function. The recurrent symptoms may have been due to stricture, or possibly to recurrent diverticulitis above the line of anastomosis.

The following case which, except for the length of the history, gave a history which strongly suggested malignant disease was treated by primary resection and anastomosis :—

E.T., a woman aged 62, was admitted to St. Mark's under my care on January 4th, 1921. On and off for ten years she had suffered from attacks of diarrhœa, coming on every two or three days, frequently commencing at night, accompanied by general abdominal pain of a griping character which lasted for about two hours. She complained of a purulent and offensive discharge from the rectum, which was sometimes slightly bloodstained. There was no vomiting or abdominal distention. She had been losing weight for some months. On examination of the abdomen there was increased resistance and some tenderness in the left iliac fossa and a sausage-shaped swelling could be felt. The rectum was normal. On sigmoidoscopy a small polypus was seen at 7½ inches, but up to 12 inches there was no evidence of tumour or stenosis. When the abdomen was opened the upper part of the pelvic colon was found to be adherent to the parietal peritoneum. The middle of the pelvic loop was much thickened and the appendices loaded with fat. When the loop was brought outside many diverticula were felt. Some of these showed signs of peri-diverticulitis and some obviously contained fæcal concretions. The pelvic colon was delivered outside, and a lateral anastomosis performed on either side of the involved area and the remainder of the pelvic loop then resected. She made an uninterrupted recovery, and was completely cured of her diarrhœa and offensive discharge. She gained weight rapidly, and has remained in excellent health ever since. Her weight on admission, January, 1921, was 9 st. 7 lb., and her weight in January, 1922, when shown at the Royal Society of Medicine, was 12 st. 7 lb.

I will refer to one other case because it illustrates another method of treatment—appendicostomy.

A man aged 70 was admitted to hospital with a tender mass in the left iliac fossa and considerable difficulty with his bowels. I explored and found well-marked diverticulitis with many adhesions. The patient was a bad subject for operation. Colostomy, owing to adhesions, presented difficulties. I decided on appendicostomy with a view to obtaining relief by regular irrigation. Unfortunately, the patient developed bronchitis after the operation and succumbed.

It will be clear from the history of the cases already described that the clinical symptoms of peridiverticular fibrosis with stenosis are not easy to distinguish from those of carcinoma. Most observers lay considerable stress on the absence of blood, but too much weight must not be given to this point. In three of the cases which I have recorded blood was occasionally noted in the stools

To my mind the most important clinical distinction is the appearance of the patient. When carcinoma of the colon has developed to a stage which brings the patient to a doctor usually with semi-obstructive symptoms the patient looks the part—he is losing weight, he looks ill.

Cases of diverticulitis usually come with a long history of capricious actions of the bowels associated with pain and tenderness in the left iliac fossa, with periods of complete freedom from symptoms. They are well covered and do not complain of losing weight. There is no suggestion of "the shadow of malignancy" in their appearance. A tumour may often be felt in the left iliac fossa of considerable size. A carcinoma of the pelvic colon, on the other hand, is usually too small to be felt (except under an anæsthetic with bimanual examination).

Pain and tenderness in the iliac fossa are the exception in carcinoma.

When there is marked inflammation in diverticulitis in addition to pain and tenderness there may be irregular pyrexia and leucocytosis and often irritability of the bladder.

Inflammatory strictures involving the pelvic colon apart from the rectum do not often occur. When they do occur they are usually low enough to be recognised with the sigmoidoscope, and may be distinguished from diverticulitis by the loss of mucous membrane at the site of stricture.

The stricture will be tunnel-like rather than ring-like as in carcinoma. The specimen I now show you is an example of non-malignant stricture of the pelvic colon which I excised from a man aged 43 in 1919. The symptoms and sigmoidoscopic appearances were in favour of carcinoma, and at the operation I could not determine whether I was dealing with an inflammatory or a malignant condition.

The microscope shows no evidence of carcinoma. The man remains well.

Before diverticulitis became generally recognised sigmoiditis and pericolitis were familiar terms, and no doubt many cases of inflammatory stricture due to diverticulitis were described under these headings.

In summarising the treatment of chronic cases of diverticulitis associated with fibrosis and stenosis I should like to sound a note of warning. I have referred to cases in which I have resected the involved portion of the pelvic colon and the patients have survived the procedure, but it will, in all probability, be a long time before I get another opportunity for a safe excision. In the great majority of cases which call for surgical treatment (apart from acute cases) marked inflammation, extensive adhesions, excess of fat in the mesentery, and a small amount of healthy colon below the affected area will all render resection, at any rate as a primary procedure, a most hazardous undertaking. In these resections three separate methods were employed: a one-stage method, *i.e.* (*a*) resection and anastomosis in one stage (an exceptionally favourable case); a two-stage method, *i.e.* (*b*) primary anastomosis with secondary resection; a three-stage method, *i.e.* (*c*), stage 1—eversion of the growth outside the abdomen, stage 2—excision and colostomy, stage 3—anastomosis.

Without doubt resection and anastomosis is the ideal procedure under the most favourable conditions. In practice we must, more often than not, be content with a temporary colostomy in the hope that, as is often the case, the inflammatory condition will subside, when closure of the colostomy may be considered.

If the mesentery of the pelvic colon is long, the involved pelvic loop may be brought outside at the time of the colostomy and dealt with as described above.

The above methods apply to those cases which have caused some measure of obstruction or which by reason of stenosis threaten to do so.

Milder types which, while causing much inconvenience do not seem to justify a colostomy at the time of operation, or in which as sometines happens a colostomy has been refused by the patient before operation, may be dealt with by invagination of dangerous looking saccules and by omental grafts overlapping the involved area. This latter procedure should be borne in mind in dealing with acute perforation. In some cases it may be wise to combine invagination or grafting with appendicostomy.

It is an interesting feature of these cases that active symptoms are often intermittent. Cases come under observation with long histories of periodic attacks followed by periods of immunity or comparative immunity from symptoms.

In the early stages of carcinoma of the colon similar intermissions are sometimes met with; but such histories are short, and are soon curtailed by the more serious and more constant local symptoms and by the "shadow of malignancy" which hovers round the victims.

The ebb and flow of chronic diverticular inflammation is doubtless dependent on variations in resistance, and due to factors other than mechanical.

In the varying toxic states associated with oral sepsis there is, perhaps, some parallel. We know that periodic bouts of sciatica, lumbago, tonsillitis, etc., have so often been permanently relieved by the discovery and treatment of a septic focus in a tooth socket or an accessory sinus as to establish without doubt a relationship between the two conditions. We may assume similar factors at work in connection with the septic colon pockets to account for similar intermissions in their constitutional effects and corresponding local changes.

After a colostomy has been performed for obstructive symptoms the surgeon may be tempted to close the colostomy if he finds that the stricture subsequently disappears. If he does, he must remember that relapses are common, and in the absence of a short circuit or colostomy are the rule rather than the exception.

I have not considered treatment of these cases on medical lines, but it is a point on which I hope to obtain information to-night.

I do not propose to consider diverticulitis of the cæcum and appendix, though I have a fine specimen of cæcal diverticulitis to show you, nor will time permit me to discuss the acute manifestations which present many interesting features. One of the most striking of these is that in some instances the symptoms have been *right-sided* instead of left, when it has been found that the pelvic colon has been drawn over to the right by adhesions, just as occasionally appendix symptoms are left-sided.

It used to be thought that the passage of wind and fæces by the bladder meant a recto-vesical or vesico-colic fistula of malignant origin. In recent years our knowledge of the sequelæ of diverticulitis has revealed that vesical fistulæ are not uncommon, and further investigation has shown that many of these fistulæ hitherto described as malignant are in reality inflammatory and secondary to inflammation around colon diverticula.

Fæcal fistulæ at the umbilicus and following a left iliac abscess may very often be laid at the door of pelvic diverticulitis.

I have not attempted to give you an historical survey nor yet a complete clinical picture of the many manifestations that are met with in association with diverticula of the colon. You will see by the table which Maxwell Telling drew up that this would involve a series of lectures. I have endeavoured rather to give you my personal experiences and to dwell on the type of case which simulates malignant disease. You will realise from what I have said that in the past many apparent cures following colostomy for supposed inoperable carcinoma of the colon should have been grouped under this heading.

## Figures and Tables

**Fig. 1. f1:**